# Evidence-based long term interventions targeting acute mental health presentations for children and adolescents: systematic review

**DOI:** 10.3389/fpsyt.2024.1324220

**Published:** 2024-03-06

**Authors:** Brigitte Gerstl, Bright Opoku Ahinkorah, Thomas P. Nguyen, James Rufus John, Patrick Hawker, Teresa Winata, Febe Brice, Michael Bowden, Valsamma Eapen

**Affiliations:** ^1^ Academic Unit of Infant, Child, and Adolescent Psychiatry Services (AUCS), South Western Sydney Local Health District and Ingham Institute, Sydney, NSW, Australia; ^2^ Discipline of Psychiatry and Mental Health, School of Clinical Medicine, University of New South Wales (UNSW), Sydney, NSW, Australia; ^3^ School of Public Health, University of Technology Sydney, Sydney, NSW, Australia; ^4^ Mental Health, School of Medicine, Western Sydney University, Sydney, NSW, Australia; ^5^ Infant, Child and Adolescent Mental Health Service (ICAMHS), South Western Sydney Local Health District, Sydney, NSW, Australia; ^6^ Child and Youth Mental Health, NSW Ministry of Health, Sydney, NSW, Australia; ^7^ Sydney Medical School, University of Sydney, Sydney, NSW, Australia; ^8^ Psychological Medicine, Sydney Children’s Hospitals Network, Sydney, NSW, Australia

**Keywords:** mental health services, child and adolescent mental health services, mental health intervention, psychological distress, evidence base

## Abstract

**Background:**

Long term intervention services have proven to be effective in improving mental health (MH) outcomes and the quality of life for children and young people (CYP).

**Aim:**

To synthesize evidence on the effectiveness of long-term interventions in improving MH outcomes for CYP, 0-17 years, presenting with MH conditions.

**Methods:**

A systematic search was carried out and the methodological quality of included long term MH intervention studies were assessed. Six databases were searched for peer-reviewed articles between January 2000 and September 2022.

**Results:**

We found 30 studies that reported on the effectiveness of a range of long-term MH interventions in the form of (i) group therapy, (ii) multisystemic behavior therapy, (iii) general services, (iv) integrated services, (v) psychotherapy, (vi) intensive intervention services, (vii) comprehensive collaborative care, (viii) parent training, and (ix) home outreach service. Among the included studies, seven were rated as high level of evidence based on the National Health and Medical Research Council (NHMRC) levels of evidence hierarchy scale and seven were of moderate quality evidence. Others were rated as lower-quality evidence. Among the studies providing high quality evidence, most were reported for group therapy, general services, and psychotherapy studies demonstrating beneficial effects.

**Conclusion:**

This systematic review provides evidence to demonstrate the benefits of a range of long-term interventions, in a range of settings, can be effective in improving MH outcomes for CYP and their families.

**Systematic review registration:**

https://www.crd.york.ac.uk/prospero/, identifier CRD42022323324.

## 1 Introduction

Mental health (MH) conditions comprise a broad spectrum of disorders affecting a child and young person’s (CYP) mood, thinking, and behavior. These conditions include chronic and long-term disorders that require sustained interventions and support, addressing the unique challenges faced by CYP over an extended period. MH problems are prevalent among CYP worldwide with 8.8% of CYP having received diagnoses for diverse mental health conditions, imposing a significant burden on public health (1). Available evidence suggests that most common MH disorders in CYP include anxiety, conduct, major depression and mood disorders, and attention-deficit/hyperactivity disorder (ADHD) among others (2–4). Thus, the escalating global prevalence of MH disorders highlight the urgent need for enhanced prevention and treatment strategies.

Acute MH needs in CYP present is characterized by significant and distressing symptoms that require prompt attention and targeted interventions. Acute presentations may include psychosomatic symptoms such as headaches, abdominal pain or discomfort associated with altered bowel function, fatigue, and chronic functional gastrointestinal disorders (such as irritable bowel syndrome) (5). These symptoms, particularly prevalent among school-aged children, significantly contribute to the overall burden of MH challenges (6) They often serve as early indicators of acute distress and require careful consideration in the context of intervention strategies. Epidemiological studies on childhood headaches from around the world report widely variable prevalence rates, with an estimated median frequency of headaches in children and adolescents at 58.4%, ranging from one-month to a lifetime prevalence (7, 8). Approximately 31.5% of children in primary and secondary schools experience headaches twice a month or more over a three-month period (9).

Attention Deficit Hyperactivity Disorder (ADHD), as a prevalent and persistent developmental disorder, exemplifies the need for acute interventions. Characterized by excessive inattention, hyperactivity, or impulsivity, ADHD becomes particularly discernible in the school-age years (10).

To address acute MH needs, it is important to delve into the prevalence of acute presentations, the potential consequences if untreated, and common categories of interventions. Recent international meta-analyses have highlighted increased rates of MH conditions among CYP, emphasizing the urgency of addressing acute presentations (11, 12). The Covid-19 pandemic further emphasized the significance of acute MH interventions, with CYP facing heightened risks during lockdowns, disruptions to education, and increased exposure to stressors (13–17).

There exists a substantial body of research linking poor MH to increased morbidity (12, 13), which can markedly impair quality of life and promote susceptibility to various physical and psychological health issues (18). Furthermore, MH problems are the leading cause of disability among young people (YP) globally, accounting for 45% of disability-related years lost (19). MH issues, if untreated, have the potential to significantly disrupt the wellbeing of children within their homes, schools, and communities. There are several interventions available for addressing MH challenges among CYP. These interventions span psychosocial programs, school-based initiatives, and parent and family-focused strategies, including psychoeducation, skills training, and cognitive-behavioral therapy (CBT). The inclusion of these varied approaches reflects the diverse landscape of interventions aimed at enhancing the mental well-being of CYP. Without proper intervention, CYP facing MH challenges are more prone to academic struggles, involvement with the criminal justice system, reliance on social services, and, in severe cases, the risk of suicide (20, 21). Studies have shown that severe MH issues, including self-harm and suicide among YP (21, 22) are difficult to treat once they have been established. This in turn can cause significant ongoing impacts well into adulthood (23). Hence, it is critical to have systems in place for early identification and treatment of MH conditions to prevent persistence into adulthood.

Besides ongoing MH morbidity for CYP, there is also a significant economic burden on the healthcare system due to high rates of hospital admissions, Emergency Department (ED) presentations, and re-presentations (24, 25). It is estimated that one in seven (14%) CYP aged between 4 and 17 years in Australia (2, 3) have a MH disorder with a recent Australian study estimating the annual incremental healthcare costs related to MH for CYP aged 4-17 years to be $AU234 million (26). In response to the growing demand for MH care among CYP, various preventive interventions, including school-based programs, have been implemented across different settings, yielding positive impacts on the wellbeing of CYP by reducing depression and anxiety through the provision of coping strategies and interventions (27). Moreover, a broad range of intervention approaches, including psychosocial interventions, have been successfully implemented in a variety of health and community settings (22, 28–32). Additionally, parent and family-focused interventions (including psychoeducation, parent and family-skills training, behavioral, psychosocial, and cognitive behavioral therapy [CBT]) are known to be efficacious in improving CYP’s MH outcomes as well as parenting behaviors and family functioning (30, 33).

Considering the prevailing burden and impact of MH disorders among CYP, it is important to identify effective interventions for MH needs. The aim of this study was to assess the effectiveness of long-term interventions specifically tailored for CYP with acute mental health needs, highlighting the need for a comprehensive understanding and addressing both immediate crises and sustained support in CYP aged 0-17 years.

## 2 Methods

This study was conducted in accordance with the Preferred Reporting Items for Systematic Review and Meta-Analyses (PRISMA) guidelines (34). A protocol for the study has been developed and registered on the Prospective Register of Systematic Reviews (PROSPERO; registration number CRD42022323324).

### 2.1 Search strategy and study selection

This systematic review utilized six electronic databases to extract studies reporting on the effectiveness of long term (definition below) interventions for MH presentations of CYP aged 0 to 17 years. Articles were searched with date restrictions from January 2000 to March 2022. Articles were limited to the English language. Duplicated studies were removed using Endnote and again in Rayyan software for systematic reviews (35).

The following databases were searched: (1) PubMed; (2) Ovid PsycINFO; (3) Web of Science, via Clarivate; (4) EMBASE; and (5) Cochrane Library. We also searched the reference lists of included studies to identify further relevant literature. The full search strategy is included in **Supplementary 1**.

### 2.2 Selection criteria

The inclusion criteria were as follows: (1) the study reported on the effectiveness of a long-term intervention on MH outcomes for CYP presenting with an acute MH condition. In this review, ‘acute mental health needs’ were defined as instances where a CYP experienced a sudden decline in their MH, often accompanied by significant distress and impaired functioning that required urgent medical attention or intervention in any setting, i.e., hospital or ED). Additionally, long-term interventions were defined as treatment for MH concerns in CYP that lasted for more than eight weeks or involved more than three visits; (2) the age of participants ranged between 0 to 17 years, or where the age range was not reported, the participant’s mean age was less than 18 years; and (3) studies published in the English language.

The following studies were excluded: (1) participant age criteria were not met (participants > 18 years of age); (2) participants were not presenting/attending a long-term intervention for the treatment of a MH condition, (3) the study focused solely on a short-term school-based programs or residential programs that did not meet the definition of long-term interventions; (4) the study focused exclusively on short-term parent-focused interventions, such as shorter duration parent peer support programs, that did not meet the criteria for long-term interventions; (5) the study focused on interventions provided exclusively through justice systems or child welfare systems; (6) the study was a quantitative meta-analysis of published literature, editorial, or scoping review; and (7) the study was not published in English.

### 2.3 Data extraction and synthesis

Four reviewers (FB, BG, PH, JRJ) independently performed initial title and abstract screening. Two reviewers (FB, PH) independently conducted full-text screening and compared results. A fifth reviewer (TW) was available to resolve or moderate any disagreements about the included articles. Four reviewers (FB, BG, PH, BOA) performed data extraction for the included articles and retrieved full-text reports using a data collection tool.

### 2.4 Quality assessment

**Table 1** provides a comprehensive overview of each of the included studies, with detailed descriptions of the studies, NHMRC levels of evidence and risk of bias, interventions examined, and the results reported. Among included studies, 7 studies were rated as providing high level of evidence based on the NHMRC levels of evidence hierarchy scale (36–42) followed by 7 studies which were rated as moderate quality evidence (43–49), and 16 articles were of lower quality (50–65). There was considerable variability between study methodologies with nearly half (46%, n=14/30 studies) of the studies used a control group to compare outcomes (36–49) (see **Table 1**). Where high quality RCT studies were reported, we only present these studies under each of the sub-headings as per the type of interventions (see **Table 1**).

**Table 1:** Characteristics of long-term intervention studies (n=30 studies).

Author	Year	Country	NHMRC level of evidence and risk of bias	Study design	Sample size	Mean age	Gender	MH symptoms	Intervention strategies	Outcomes
Asarnow et al. (36)	2009	USA	Evidence: High (Level: I) Level of bias: Moderate	Randomised Controlled Trial (RCT)	INT n=211 CNT n=207	INT 17.3 years (age range: 13-17 years)	INT Male: 45/211 Female: 166/211 CNT Male: 47/207 Female: 160/207	INT Depression and mood disorders (n=93/211) CNT Depression and mood disorders (n=85/207)	• Integrated services • General services • Intensive intervention services • Comprehensive collaborative care and medication management • CBT • Individualised treatment or therapy	The intervention ( clinical team management, CBT, medication, referral, and follow-up), compared with treatment as usual improved outcomes, by reducing severe depression at 6 months post intervention. Over an 18-month follow-up period, post intervention, a decrease in rates of depression, and improved mental health-related quality of life was observed.
Boege et al. (37)	2021	Germany	Evidence: High (Level: I) Level of bias: Moderate	RCT	INT n=54	INT 13.5 years	INT Male: 25/51 Female: 28/51 CNT Male: 23/47 Female: 24/47	INT Internalising disorders, conduct disorders/ADHD(n=20/51) CNT Internalising disorders, conduct disorders/ADHD(n=/47)	• Intensive intervention services In-patient treatment • General services • Individualised treatment or therapy • Comprehensive collaborative Care or medication management • Group therapy • Occupational therapy • Crisis intervention	Post intervention ( in-patient stay followed by 12 weeks of home treatment) CGAS scores increased on clinical functioning (62.44 versus 69.82) and LOS as an in-patient reduced to 47.65 days. HoNOSCA scores decreased significantly from pre-intervention (T2) throughout the intervention (T3) and post intervention (T4)
Lipman et al. (38)	2006	Canada	Evidence: High (Level: I) Level of bias: High	RCT	INT n= 62 CNT n= 61	INT 9.3 years (age range: 7-11 years) CNT 9 years (age range: 7-11 years)	INT Male: 50/62 Female: 12/62 CNT Male: 52/61 Female: 9/61	INT Anger or aggressive behaviours(n=62/62) CNT Anger or aggressive behaviours (n=61/61)	• Integrated services • Group therapy • General services	Post intervention, ( parent/caregiver psychoeducation group classes, weekly child group sessions, and in-home family practice sessions) non-significant reductions in CYP anger , hostility , aggression and externalizing behaviour and parenting stress were observed.
Ogden et al. (39)	2006	Norway	Evidence: High (Level: I) Level of bias: Moderate	RCT	INT n= 46 CNT n=29	15.1 years	Male: 48/75 Female: 27/75	INT Behaviour disorders (n=46/46) CNT Behaviour disorders (n=29/29)	• Behaviour therapy	Multisystemic Treatment (MST) (included intensive home- and community-based intervention) was effective in reducing out of home placement and behavioural problems. Post MST there was less delinquent behaviours compared with CYP that received TAU.
Petrenko et al. (40)	2017	USA	Evidence: High (Level: I) Level of bias: High	RCT	INT n=16 CNT n=14	INT 6.5 years (age range: 4-8 years) CNT 6.6 years (age range: 4-8 years)	INT Male: 10/16 Female: 6/16 CNT Male: 13/14 Female: 1/14	INT Fetal alcohol syndrome/prenatal fetal alcohol syndrome (n=8/16) CNT Fetal alcohol syndrome/prenatal fetal alcohol syndrome (n=5/14)	• Group therapy • General services	Medium-large post-intervention improvements in child emotion regulation, anxiety and self-esteem compared to the TAU group.
Richardson et al. (41)	2014	USA	Evidence: High (Level: I) Level of bias: Low	RCT	INT n= 50 CNT n=51	INT 15.1 years (age range: 13-17 years) CNT 15.5 years (age range: 13-17 years)	INT Male: 14/50 Female: 36/50 CNT Male: 14/51 Female: 37/51	INT Depression and mood disorders (n=50/50) CNT Depression and mood disorders (n=51/51)	• Comprehensive collaborative Care or medication management • CBT	12-month follow-up post intervention of CYP who were randomized to the intervention group ( in-person engagement session and regular follow-up by clinical team) using the Child Depression Rating Scale–Revised (CDRS-R) showed a significant decrease in depression scores compared with the control group that received TAU. The clinical rate for depression among CYP in the intervention decreased from 67.6%vs 38.6%, with a remission rate difference of 50.4% versus 20.7%.
Van Den Hoofdaker et al. (42)	2007	Netherlands	Evidence: High (Level: I) Level of bias: Moderate	RCT	INT n= 47 CNT n= 47	7.4 years (age range: 4-12 years)	Male: 76/94 Female: 18/94	INT ADHD: 48/48 CNT ADHD: 48/48	• Behavioural parent training	CYP in the intervention group that received behavioural parent training (BPT) ( 12 group sessions with each lasting 120 minutes) indicated a significant improvement in both ADHD internalising and externalising behaviours. There were no significant differences in ADHD symptoms and in levels of parenting stress.
Haft et al. (43)	2019	USA	Evidence Moderate (Level: III-2) Risk of bias: Moderate	Cohort	INT n=99 CNT (non-mentored youth) n = 51 CNT (typically-developing youth) n = 84	INT 12.0 years (age range: 8-16 years) CNT (non-mentored youth) 12.1 years (age range: 8-16 years) CNT (typically-developing youth) 11.5 years (age range: 8-16 years)	INT Male: 59/99 Female: 40/99 CNT (non-mentored youth) Male: 28/51 Female: 23/51 CNT (typically-developing youth) Male: 45/84 Female: 39/84	INT ADHD (n=99/99) CNT (non-mentored youth) ADHD (n=51/51)	• Peer support or problem-solving	CYP in the Eye-to-Eye intervention group (peer monitoring by middle school youth of elementary school children with ADHD) showed significant decreases in depression and self-esteem compared with children that did not receive peer mentoring.
Lilly et al. (44)	2020	USA	Evidence Moderate (Level: III-2) Risk of bias: High	Cohort	INT n=483 CNT n=283	INT 9.7 years (age range: 1-17 years) CNT 9.5 years (age range: 1-17 years)	INT Male: 234/483 Female: 249/483 CNT Male: 145/283 Female: 138/283	INT Behaviour health problems(n=483/483) CNT Behaviour health problems(n=283/283)	• Integrated services	CYP who used the intervention hub clinics (where patients receive behavioural health primary care physician (PCP) consultations in a co-located setting) were more likely to schedule an appointment with a primary health care physician compared with CYP in the extension clinic group (PCP on-site only with referrals to behavioural health providers in hubs) who were less likely to engage with a primary care physician (92.3% versus 78.1%). CYP in the intervention group were also 2.4 times more likely to cancel a scheduled appointment with their primary health care physician and 2.2 times more likely to not turn up to a scheduled appointment compared with CYP in the extension clinic patients.
Shippee et al. (45)	2018	USA	Evidence Moderate (Level: III-2) Risk of bias: Moderate	Cohort	INT n= 162 CNT n= 499	INT 14.9 years (age range: 12-17 years) CNT 14.8 years (age range: 12-17 years)	INT Male: 31/162 Female: 131/162 CNT Male: 157/499 Female: 342/499	INT Depression and mood disorders: 162/162 CNT Depression and mood disorders: 499/499	• Intensive intervention services • Individualised treatment or therapy • Group therapy • CBT	CYP in the EMERALD (Early Management and Evidence-based Recognition of Adolescents Living with Depression) intervention group reported significantly greater rates of depression remission and treatment response compared with the control group which had received TAU.
Cordell et al. (46)	2017	USA	Evidence Moderate (Level: III-2) Risk of bias: Moderate	Cohort	INT n=17878 CNT n=542647	–	INT Male: 10230/17878 Female: 7648/17878 CNT Male: 314117/542647 Female: 228530/542647	INT Mental health disorders (not specified) (n=17878/17878) CNT Mental health disorders (not specified) (n=542647/542647)	• Integrated services • Wellness therapy • Peer support or problem-solving • Substance abuse counselling • General services	CYP in the intervention group (multidisciplinary case management team for social and MH services) showed a significant reduction in the use of crisis services post intervention compared to the control group (receiving TAU) where crisis service use continued to increase highlighting the benefits associated with the intervention.
Enns et al. (47)	2017	Canada	Evidence Moderate (Level: III-2) Risk of bias: Moderate	Cohort	INT n=485 CNT n=1884	No mean age provided Age range: 5-17 years	INT Male: 401/485 Female: 84/485 CNT Male: 1604/1884 Female: 280/1884	INT ADHD (n=485/485) CNT ADHD (n=1884/1884)	• Behaviour therapy	CYP in the intervention group (which included individual therapy, parent support, group therapy, education, and medication management with a multidisciplinary team) had higher rates of medication use and adherence for ADHD treatment compared to CYP in the control group post-intervention. There were no differences in rates of hospital or child welfare service presentations.
Fjermestad et al. (48)	2020	Norway	Evidence Moderate (Level: III-2) Risk of bias: Moderate	Cohort	INT (targeted prevention sample) n=82 CNT (specialist clinical sample) n=88	INT: 11.6 years (age range: 8-16 years) CNT (specialist clinical sample) 11.7 years (age range: 8-16 years)	INT Male: 20/82 Female: 62/82 CNT Male: 40/88 Female: 48/88	INT Not specified CNT Anxiety (n=88/88)	• CBT	The ‘FRIENDS for life’ intervention program (10-session CBT program), found anxiety symptoms significantly reduced post-intervention in both the targeted prevention sample where less-specialized school nurses administered the treatment compared with the specialist clinic sample.
McDonell et al. (49)	2010	USA	Evidence Moderate (Level: III-2) Risk of bias: High	Cohort	INT n= 106 CNT N=104	INT 15.5 years (age range: 12-17 years) CNT 15.3 years (age range: 12-15 years)	Male: 89/210 Female: 121/210	INT Oppositional defiant disorder (n=86/106) Conduct disorders (n=75/106) CNT Not reported	• DBT	In the 12-month follow-up period, there were significant improvements in overall functioning, decreased number of psychotropic medications used as well as non-suicidal self-injury in the DBT intervention group compared to the historical control group (received TAU).
Baruch et al. (50)	2011	England	Evidence: Low (Level: IV) Risk of bias: High	Pre- and post-study	INT n=123	INT 14 years (age range: 10-17 years)	INT Male: 71/123 Female: 52/123	INT Antisocial behaviour (n=120/123)	• Behavioural parent training • Group therapy	The intervention (six, 2-hour classes that cover parent teen interaction, behavioural contracts, appropriate consequences for high-risk challenging behaviour, praising the teenager, nurturance strategies, and how to enlist and use outside support) showed significant decreases post-treatment for internalising problems, externalising problems and total problems on the CBCL.
Clossey et al. (51)	2018	USA	Evidence: Low (Level: IV) Risk of bias: High	Pre- and post-study	INT n=29	INT 11 years (age range: 5-17 years)	INT Male: 17/29 Female: 12/29	INT Behaviour disorders (n=29/29)	• Family therapy	Family function using the Child Outcomes Survey (COS) 3 months post-intervention (Rapid Response Program or Behavioral Health Rehabilitation Service which included family therapy) showed improved family functioning scores from baseline as well as improved child function scores.
Duffy et al. (52)	2014	Scotland	Evidence: Low (Level: IV) Risk of bias: High	Pre- and post-study	INT n=113	INT 14.9 years (age range: 7-17 years)	INT Male: 38/113 Female: 75/113	INT Mental health disorders (not specified) (n=103/103)	• Intensive intervention services • Family therapy • Group therapy • Comprehensive collaborative Care or medication management • Integrated services • General services	Following CAMHS Intensive Treatment Service (Intensive Treatment Service), CGAS ratings significantly improved by 16 points and HoNOSCA scores significantly decreased by a mean of 6.94 points. There were also significant improvements in self-reported depression, anxiety and anger ratings post-intervention.
Evans et al. (53)	2008	USA	Evidence: Low (Level: IV) Risk of bias: Moderate	Pre- and post-study	INT n=159	INT 11.7 years	INT Male: 94/159 Female: 65/159	INT Mental health disorders (not specified) (n=159/159)	• CBT • Family therapy	Following implementation of a school-linked model of mental health prevention and treatment (CBT, skills-building, interpersonal therapy) for children and their families, children who had less psychopathology and a higher level of clinician-rated functioning prior to the intervention were more likely to successfully complete treatment. Parental involvement in therapy was significantly associated with a longer duration of CYP treatment, and an increase in missed appointments.
Flynn et al. (54)	2019	Ireland	Evidence: Low (Level: IV) Risk of bias: High	Pre- and post-study	INT n=84	INT 15.7 years (age range: 13-18 years)	INT Male: 12/84 Female: 72/84	INT Self-harming behaviours (n=84/84)	• DBT	CYP who received DBT as part of the intervention reported significant improvements in depression, borderline symptoms, trait anger and suicidal ideation post-intervention and at 16 week follow up. Total ED presentations and acute inpatient admissions also reduced on both follow-up periods.
Fox et al. (55)	2009	USA	Evidence: Low (Level: IV) Risk of bias: High	Pre- and post-study	INT n=102	INT 2.7 years (age range: 1-5 years)	INT Male: 59/102 Female: 43/102	INT Oppositional defiant disorder: (n=81/102)	• Parent-child dyad therapy	Parents and children who participated in the intervention (parent management training) had significantly improved parent-child interactions post-intervention and significantly reduced the frequency and severity of behavioural problems in the child.
Griffiths et al. (56)	2017	England	Evidence: Low (Level: IV) Risk of bias: High	Pre- and post-study	INT n=302	INT Age range: 11-22 years	INT Male: 109/302 Female: 193/302	INT Mental health disorders (not specified) (n=161/161)	• Integrated services	There were significant improvements in quality of life, anxiety and depressive symptoms across a 2-year intervention period (Adolescent Mentalization-based Integrative Treatment).
James et al. (57)	2011	England	Evidence: Low (Level: IV) Risk of bias: High	Pre- and post-study	INT n=25	INT: 15.5 years (age range: 13-17 years)	INT Male: 3/25 Female: 22/25	INT Borderline personality disorder (n=25/25)	• DBT	CYP who had completed DBT treatment reported significant reductions in depression, hopelessness and were less likely to self-harm whilst global functioning significantly improved.
Jaycox et al. (58)	2003	USA	Evidence: Low (Level: IV) Risk of bias: Moderate	Pre- and post-study	INT n=1088	INT 15.8 years (age range: 12-19 years)	INT Male: 838/1088 Female: 250/1088	INT Substance abuse disorders (n=1088/1088)	• Substance abuse counselling • Integrated services	Following the intervention (substance abuse treatment program), reported mental distress was reduced. The proportion of those reporting severe mental health problems only reduced slightly 3 months post-intervention.
Lu et al. (59)	2021	Australia	Evidence: Low (Level: IV) Risk of bias: High	Pre- and post-study	INT n= 3098	INT 11 years (age range: 5-18 years)	INT Male: 1385/3080 Female: 1712/3080	INT Mental health disorders (not specified): 3098/3098	• General services	CYP post-intervention (community child and youth mental health services) reported substantial improvements on the HoNOSCA and CGAS with moderate improvements on the parental reported SDQ scale.
Mantzouranis et al. (60)	2018	Switzerland	Evidence: Low (Level: IV) Risk of bias: High	Pre- and post-study	INT n= 179	INT 15.8 years	INT Male: 102/179 Female: 77/179	INT Mental health disorders (not specified) (n=179/179)	• Intensive intervention services • Integrated services	An assertive community treatment program offered by multidisciplinary team (composed of psychiatrists, nurses, and social workers) showed improved outcomes on HoNOSCA and Global Assessment of Functioning Scale (GAF) from admission to discharge. CYP showed an overall clinical improvement of 7% at discharge to functional levels.
Newman et al. (61)	2014	England	Evidence: Low (Level: IV) Risk of bias: High	Pre- and post-study	INT n= 24	INT 12.8 years (age range: 8-17 years)	No data available on gender	INT Behaviour disorders (n= 24/24)	• Group therapy	Nonviolent Resistance (NVR) parenting groups intervention consisted of 12 weekly sessions with parents of children with aggressive, violent, or controlling behaviour. Parental Strengths and Difficulties Questionnaire (pSDQ) completed by the parent showed a significant reduction in total difficulties, total emotions, and total impact scores post-intervention.
Rickwood et al. (62)	2015	Australia	Evidence: Low (Level: IV) Risk of bias: High	Pre- and post-study	INT N = 24034	INT Age range 12-25 years	INT Male: 8965/24034 Female: 15069/24034	INT Anxiety (n=25708) Anger (n=3859) Stress (n=3521) Suicidal ideation or self harm (3834) Behavioural problems (n=1389) Eating disorders (n=1159) Psychosis (n=531) Borderline personality disorder (n=523)	• CBT	Younger males had a higher likelihood of presenting with anger and behavioural problems compared to other age- and sex-defined groups. Younger females were more likely to present for deliberate self-harm. Over one-third of clients showed significant improvement in psychological distress (measured by K10) from presentation to last assessment. Improvements in psychosocial functioning (measured by SOFAS) were also observed. 66% of clients demonstrated significant improvement on one or both measures.
Sibley et al. (63)	2016	USA	Evidence: Low (Level: IV) Risk of bias: High	Pre- and post-study	INT N = 218	INT 13.3 years	INT Male: 161/218 Female: 57/218	INT ADHD (n=218/218)	• Group	This intervention involved delivery of a secondary school based behavioural consultation model for adolescents with ADHD over one year. Consultations were coordinated successfully for some students but not others. Only 38.5% were able to sustain monthly contact between interventionists and consultants.
Simpson et al. (64)	2010	Scotland	Evidence: Low (Level: IV) Risk of bias: High	Pre- and post-study	INT n= 57	INT 15 years (age range: 11-17 years)	INT Male: 27/57 Female: 30/57	INT Depression and mood disorders (n=13/57) Self-harm/suicide ideation (n=12/57) Psychosis (n=11/57) Eating disorders (n= 11/57) Anxiety disorders (n=10/57) ASD (n=5/57)	• Integrated services • Intensive intervention services • General services • Group therapy • Family therapy	An intensive intervention comprising of a nurse-led Fife Intensive Therapy Team (FITT) providing regular consultation and psychiatric assessments and interventions for patients when required. Mean HoNOSCA scores decreased significantly at discharge.
Webster-Stratton et al. (65)	2011	USA	Evidence: Low (Level: IV) Risk of bias: High	Pre- and post-study	INT n= 78	INT 4.9 years (age range: 3-8 years)	INT Male: 58/78 Female: 20/78	INT Conduct disorder and/or oppositional defiant disorder: 78/78	• Parent-child dyad therapy	The intervention comprised of a parenting group program, which consisted of 12 weekly, 2-hour sessions. Follow-up 8-12 years later showed reductions in conduct problems in adolescence. Post-treatment parent-child coercion and externalizing problems were associated with worse outcomes in adolescence.

Further details describing *quality assessment, data extraction and Risk of Bias* can be found in **Appendices 1-3**.

## 3 Results

This evaluation included identifying effective interventions (mechanisms), their outcomes, and the specific settings and locations (context) in which the interventions were implemented (66).

### 3.1 Study selection

**Figure 1:**
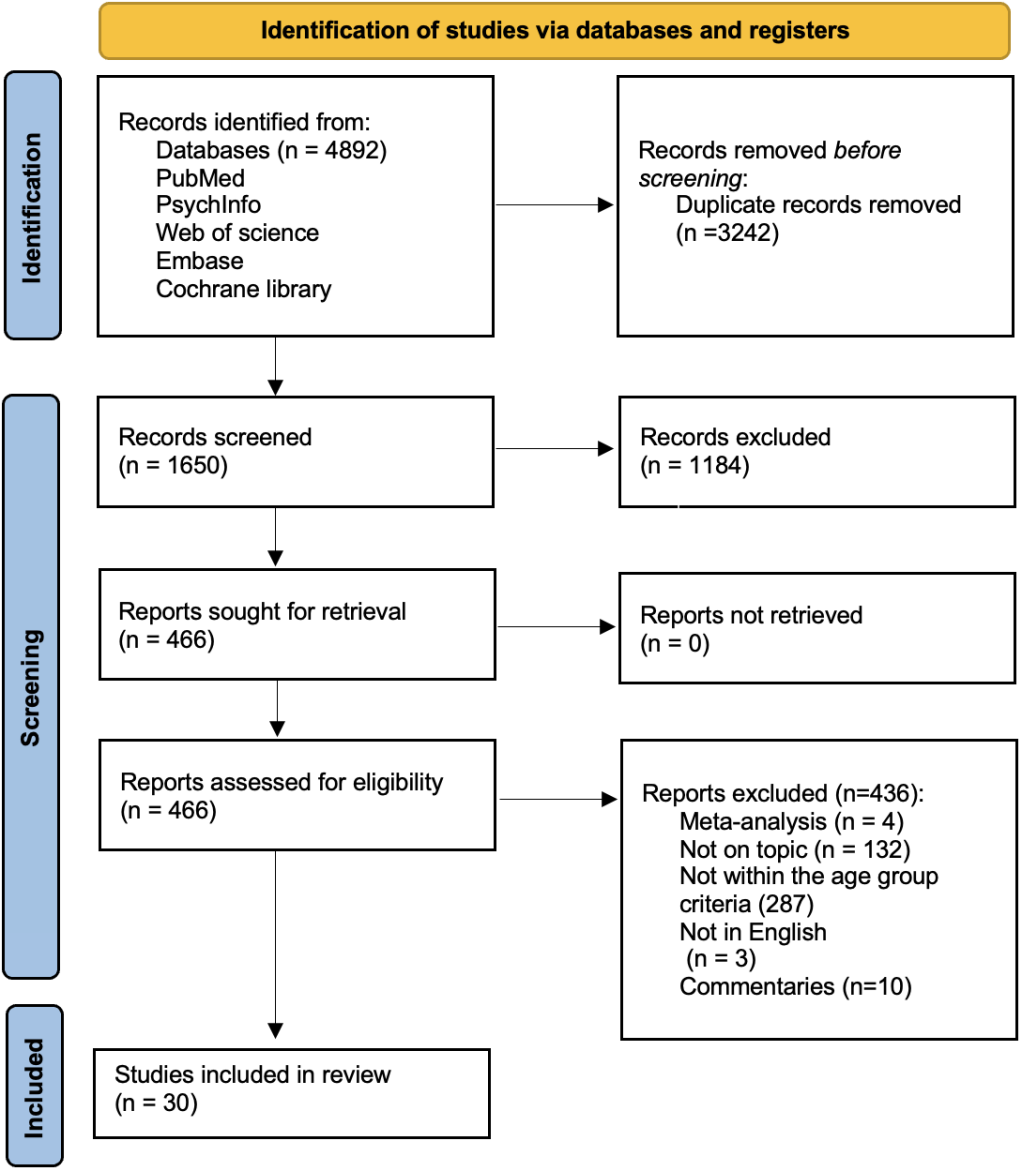
Prisma statement.

Our initial search yielded 4,892 results, of which 3,242 were removed as duplicates, resulting in 1,650 articles that were then reviewed. After title and abstract screening, 436 potential studies were assessed for eligibility of full-text screening, and 30 studies met the eligibility criteria. The systematic review process, using the PRISMA flowchart, is depicted in **Figure 1**.

### 3.2 General characteristics of the included studies

**Table 2** summarizes characteristics of the included studies. Studies were conducted in various geographical regions; including the USA (n=14) (36, 40, 41, 43–46, 49, 51, 53, 55, 58, 63, 65), England (n=4) (50, 56, 57, 61), Canada (n=2) (38, 47), Australia (n=2) (59, 62), Norway (n=2) (39, 48), Scotland (n=2) (52, 64), Germany (n=1) (37), Netherlands (n=1) (42), Ireland (n=1) (54), and Switzerland (n=1) (60).

**Table 2:** Demographic characteristics of participants from the included studies.

	Intervention n (%)	Control n (%)
Number of studies	30	14
Participants	46,338 (7.8)	546,151 (92.2)
Gender		
Female	11,095 (44)	229,569 (42)
Male	14,113 (56)	316,212 (58)
Age		
Mean age (years) (SD)	12.5 (3.7)	12.6 (3.6)
Mean age group (years) (SD)		
0-2	–	–
3-5	2 (6.7)	–
6-11	7 (23.3)	5 (35.7)
12-17	21 (70)	9 (64.3)
Ethnicity		
White/Caucasian	5742 (24)	67719 (12)
Black/Africa American	1,905 (8)	56478 (10)
Latino/Hispanic	8,761 (37)	246,796 (45)
Asian	2 (0.01)	2 (0.0004)
First Nations/ATSI	243 (1)	–
Mixed/Other	6,899 (29)	172677 (32)

ATSI, Aboriginal and Torres-Strait Islander; SD, Standard deviation.

- No data available.

### 3.3 Study findings

**Effect of interventions on mental health symptoms**

Using a narrative approach, we categorized intervention types according to the following sub-headings described in this section (**Table 1**).

#### 3.3.1 Group therapy

There were six studies (20%) (RCT (n=1) (38), pre- and post (n=4) (51–53, 64), cohort (n=1) (47) studies) that reported on group therapy interventions. The number of group sessions ranged from between 8-12 sessions over a duration of 13-30 weeks. An RCT (38) of high quality NHMRC evidence, although at a high level of risk of bias, used an adapted, manual-based group CBT program where children were taught problem-solving techniques to manage their temper. The intervention also included parent/caregiver psychoeducation/skill-building group sessions and three in-home family practice sessions to support primary school aged CYP (aged between 7 to 11 years of age) and their families. The RCT found no significant differences and small effect sizes between both groups for all primary outcomes (e.g., child anger and child aggressive behavior). However, the magnitude of improvement (difference scores) was greater in the intervention group on parent-rated measures such as Child Behavior Questionnaire, Parenting Stress Index-Short Form (PSI-SF), compared with the control group (38).

#### 3.3.2 Multisystemic behavior therapy

An RCT study (39), of high-quality NHMRC evidence with moderate risk of bias examined the effectiveness of a multisystemic treatment (MST) in reducing behavior problems and preventing out-of-home placement, compared to Regular Child Welfare Services (RS). An assessment was administered at the time of entry into the study (prior to randomization) and after completion of MST treatment (average treatment time was 24.3 weeks, with a range of 7-38 weeks) or approximately 6 months after the intake of the RS group. The results indicated that MST participants were less likely to have left their home compared with RS youth. About 72% of MST and 52% of RS youth lived at home or were under supervision at follow-up. Additionally, YP in the MST group also scored significantly lower on the Self-Report Delinquency Scale (SRD) compared with youth who received RS (39).

#### 3.3.3 General services

General services included psychiatric evaluations and treatment, regular review of MH treatment plans, psychosocial treatment and psychoeducation for families and parents. Among the reviewed studies, eight studies (27%) [cohort studies (n=1) (46), RCT (n=3) (36, 40, 47), pre-and post-studies (n=4) (52, 59, 62, 64)] reported on general services. The duration of general service interventions ranged from 4-10 months. CYP aged between 6-11 years, who engaged with general service interventions had ADHD (47), anger (38), aggression (38) or fetal alcohol spectrum disorders (FASDs) (40). YP aged 12-17 years, receiving general services, presented with a range of MH symptoms such as anxiety (62, 64), aggression (62), ASD (42), behavioral disorders (62), borderline personality disorder (62), depression and mood disorders (36, 62, 64), eating disorders (62, 64), psychosis (62, 64), and self-harm/suicide ideation (62, 64).

One RCT study evaluated the effectiveness of the ‘Families on Track’ Program, designed for children aged 4-8 years with fetal alcohol spectrum disorder. Despite the high risk of bias, the study was considered to provide high-quality evidence. The program’s outcomes were compared to a control group that received a neuropsychological evaluation and a community referral (40). The ‘Families on Track’ program included a diagnostic and neuropsychological evaluation, as well as separate a 30-week program for children and caregivers. Children’s program aimed to develop social competence, while caregivers’ program focused on modifying parenting attitudes through CBT and motivational interviewing strategies. The intervention significantly improved emotional regulation in children, as reported by caregivers. Compared to the control group, the intervention group exhibited a medium-to-large effect size, while the control group showed a medium-sized decline (Emotion Regulation Checklist [ERC] = 1.18). Caregiver-reported self-esteem and anxiety also showed a medium-to-large group effect size (Impairment Rating Scale [IRS] self-esteem = 0.77), although statistical significance was not reached.

A high-quality RCT with moderate risk of bias assessed the long-term stability of CYP with depressive symptoms receiving home treatment (HT) versus a control group undergoing inpatient treatment-as-usual (TAU) (37). HT proved effective, with fewer intervention group patients requiring ambulatory care post-discharge (37.5% vs. 50%), indicating greater stability and reduced outpatient needs. After 4.3 years, 70% of intervention group parents were satisfied, compared to 36.8% in the control group. Furthermore, 43.7% of HT parents found the professional assistance and advice received at home valuable (37).

#### 3.3.4 Integrated services

From the synthesized studies, a total of nine studies focused on a range of integrated services (30%) (pre-and post (n=5) (44, 52, 56, 60, 64), RCT (n=1) (36), cohort (n=3) (46, 47, 58). The duration of all integrated services ranged from 15-35 weeks. CYP (6-11 years) were accessed for ADHD (47), anger (38), aggression (38), and behavioral problems (44). YP (11-17 years)received integrated interventions for adjustment disorder (46), anxiety disorders (52, 60, 64), ASD (46, 64), behavioral disorders (39), conduct disorders (46, 60), depression and mood (46, 52, 60), eating disorders (52, 64), personality disorders (60), psychosis (46, 60, 64), substance use (46, 58), and self-harm/suicide ideation (52, 64, 67).

A high-quality RCT with moderate risk of bias evaluated the outcomes of a primary care quality improvement intervention for YP (aged 13-21 years) with depression and compared to a control group that received TAU (36). The intervention, led by a clinical care manager, included consumer education, pharmacological and psychosocial treatment, CBT training, and treatment modality choice. Severe depression scores decreased at 6 months but did not significantly differ from the control group at 18 months. Recovery time was 8.76 months (SD=0.35) for the intervention group and 9.65 months (SD=0.37) for the control group (36).

#### 3.3.5 Psychotherapy

##### 3.3.5.1 Cognitive behavioral therapy

There were seven studies (23%) [pre-and post (n=5) (45, 48, 53, 57, 62), RCT (n=2) (36, 41)] that explored CBT-based programs for CYP (ages 8-16 years). Most YP (12-17 years) accessed CBT for anxiety (48, 62), aggression (62), behavioral disorders (62), borderline personality disorder (57, 62), depression and mood disorders (36, 41, 45, 62), eating disorders (62), and psychosis (62). There were two RCT studies (36, 41) that reported the effective benefits associated with CBT. One study has been discussed elsewhere given that the intervention reported involved a combination of other MH modalities (i.e., integrated services).

One RCT examined cognitive-behavioral therapy (CBT) sessions and follow-up over six months (36) and compared outcomes with a control group who had received TAU. Results indicated that YP receiving CBT recovered nearly one month earlier (8.76 vs. 9.65 months) than the control group (n=43%). Although not statistically significant, the recovery occurred almost one month (~27 days) earlier in the intervention group.

##### 3.3.5.2 Dialectical behavior therapy sessions

Three studies (7%) (49) evaluated DBT outcomes in YP (ages 12-17) with borderline personality disorder (BPD) (49), oppositional defiant disorder (ODD) (49), conduct disorder, depression, post-traumatic stress disorder (PTSD), and suicidal tendencies (40, 49, 60). A moderate-quality cohort study (n=41) demonstrated DBT’s efficacy in community MH settings. In this study, YP receiving DBT showed a significant increase in in Children’s Global Assessment Scale (CGAS) scores (mean change=14.14, p<0.001) and lower rates of suicidal/self-harm behavior (49, 54), over 12 months compared to controls (49). Limitations include the historical control group’s limited data.

#### 3.3.6 Intensive intervention services

Five studies (17%) examined intensive intervention services for CYP (ages 7-17) with various MH concerns, including anxiety (62, 64), aggression (62), ASD (64), behavioral disorders (62), depression and mood disorders (36, 45, 62, 64), eating disorders (62, 64), psychosis (62, 64), and self-harm/suicide ideation (62, 64). A community outreach program (Child and Adolescent Mental Health [CAMH]) showed a significant reduction in Health of the Nation Outcome Scales for Children and Adolescents (HoNOSCA) scores (mean change: 6.94, p<0.001) and a 65% clinical improvement in 65% of CYP (mean CGAS increase: 16.4 points, p<0.001). Another study demonstrated a substantial decrease in HoNOSCA scores (mean decrease: 10.95, p<0.001) over 23.6 weeks for CYP with mood disorders and self-harm behaviors (57). Additionally, this study also revealed that the analysis of changes using the World Health Organization Quality of Life Instrument (WHOQoL-BREF) indicated significant improvements in psychological (mean: 33.43 to 50.09), physical (mean: 53.43 to 65.26), and environment (mean: 70.0 to 77.87) functioning (64).

#### 3.3.7 Comprehensive collaborative care

Five studies (17%) examined pharmacotherapy for CYP (ages 7-17) primarily targeting depression and mood disorders (36, 41, 45, 52). A high-quality RCT demonstrated the effectiveness of a collaborative care intervention, including antidepressant medications and CBT, for youth aged 13-17 with depression (41). Patients in the intervention group showed a significant decrease in Child Depression Rating Scale-Revised (CDRS-R) scores compared to the control group at 6 months (p<0.001) and 12 months (p<0.001), indicating higher rates of response (67.6% vs. 38.6%) and remission (50.4% vs. 20.7%) (44).

#### 3.3.8 Parent training

Two studies explored the effectiveness of behavioral parent training (BPT) interventions (42, 50). One RCT study involving children diagnosed with ADHD (42), 12-week group sessions (which comprised of 2 hours of 12-week group sessions over a 5-month period) led to significant improvements in internalizing and externalizing behaviors. While ADHD symptoms and parenting stress remained stable, the reduction in behavioral challenges substantiated the intervention’s effectiveness (42). The other RCT targeted parents of YP with conduct problems and reported notable reductions in internalizing and externalising problems among their adolescent children post-intervention (intervention consisted of 12 parents who underwent six, 2-hour classes covering a range of parent-teen behaviours) (50) (**Table 1** details further information about this study). After the program, internalizing problems (Cohen’s d = 0.61) shifted from clinical to non-clinical levels (below 60), while externalizing problems (d = 0.73) remained within the clinical range. Total problem scores transitioned from clinical to borderline range (below 63) with a Cohen’s d of 0.79 (50).

Moreover, the National Longitudinal Survey of Youth (NLSY) study evaluating a parenting program for children diagnosed with Oppositional Defiant Disorder (ODD) and/or conduct disorder provided significant evidence (65). Follow-up data encompassed children aged 3-8 years. Compared with the control group, the intervention group exhibited a higher likelihood of reporting delinquent acts (84% versus 67%), with a lower frequency (42 acts versus 54 acts) (48). Additionally, the intervention group demonstrated reduced rates of sexual activity compared to the control group (21% versus 47.8%). Notably, children in the intervention group with non-clinical ECBI problem scores post-treatment displayed fewer delinquent acts in adolescence compared to those with clinical scores (t (57) = 4.52, p <.001).

#### 3.3.9 Home outreach services

A RCT study, deemed of high-quality evidence with moderate risk of bias, reported outcomes of a home treatment intervention for CYP (37). CYP in the intervention group received a shortened inpatient stay (mean = 47.7 days) where discharge was determined by stability in level of functioning. This was followed by 12 weeks of home treatment including case management, psychoeducation, and therapy sessions. and up to three appointments a week. Patients also received supportive therapies (e.g., occupational and music therapy) and had access to crisis management services. Control group CYP had standard inpatient stays (mean = 69.4 days) with similar therapies. Four-year follow-up revealed stable treatment effects in both groups. Parents of intervention CYP expressed higher satisfaction, indicating home treatment as a promising alternative to standard inpatient care (37).

## 4 Discussion

The aim of this study was to evaluate the effectiveness of long-term interventions in enhancing continuity of MH care and reducing crises among CYP. This systematic review encompassed diverse interventions in various settings for CYP facing MH conditions. To translate these findings into actionable strategies, critical considerations emerged. Considerations emerged to translate these findings into strategies, presenting a spectrum of long-term strategies. This review covered a broad spectrum of long-term interventions for CYP with MH conditions across diverse settings.

Among long term interventions presented, group therapy (i.e., anger management group therapy), general services (i.e., psychiatric evaluations), integrated services (i.e., linkage with specialty MH and community services), and various forms of psychotherapy (i.e., individual and group sessions) were commonly reported. Interventions using psychotherapy (combining therapeutic approaches such as, family therapy, DBT and CBT group sessions, and parent-child dyad sessions) provided evidence suggesting potential benefits for improving overall psychological outcomes for CYP and their families/caregivers.

Similar findings reported in this study have also been reflected in other systematic reviews by Pilling et al. (32), Piquero et al. (68) and Perderson at el (33). This study substantiates the effectiveness of parent training programs in reducing high-risk challenging behavior for CYP diagnosed with ADHD, emphasizing the effectiveness of psychotherapeutic approaches and parent training programs in managing challenging behaviors, particularly in those diagnosed with ADHD.

Evidence from this study reinforces the efficacy of CBT and family/group-focused interventions in effectively managing anger and aggressive behaviors among CYP. These results align with previous systematic reviews conducted by Das et al. (22) and Pederson et al. (33). Moreover, this study revealed a reduction in delinquent and emotional behaviors among CYP undergoing MST compared to those receiving TAU. However, despite the positive outcomes observed in this study, a systematic review by Littell et al. (65) did not find substantial evidence indicating that MST confers significant advantages over other mental health treatment interventions.

Group therapy emerged as a pivotal intervention, especially in addressing MH challenges among adolescents at high risk of suicide McCauley et al. (69) emphasized the positive outcomes of DBT within a group setting, indicating the potential impact of group therapy in complex MH scenarios. Moreover, the versatility of group therapy extends to the realm of treatment-resistant psychosis, as reported by Polese et al. (70) in their systematic review. They suggested that psychotherapy interventions, including group therapies, could be considered in the context of treatment-resistant psychosis, supporting the broader applicability of group therapy in challenging clinical scenarios.

Recognizing the collaborative and supportive environment that remains integral to group settings is important. This collaborative approach plays a role in achieving therapeutic breakthroughs, especially in challenging clinical scenarios. This review aligns with a growing body of evidence, suggesting that group therapy holds promise not only in conventional psychiatric treatments but also in situations where individuals exhibit heightened risk factors or are not suitable for standard interventions.

Furthermore, the evidence indicates that DBT interventions are effective in mitigating self-harm and suicidal ideation among YP. This aligns with outcomes reported in reviews by DeCou et al. (71), Witt et al. (72), and Hawton et al. (73), all indicating the effectiveness of DBT interventions in reducing suicide-related outcomes. These interventions have shown to decrease the necessity for crisis intervention services. Collectively, the evidence from these systematic review’s advocates for the use of DBT as a promising intervention for YP exhibiting suicidal or self-harm behaviors.

In relation to the duration of psychotherapeutic interventions, it is important to take into consideration the varied temporal scopes inherent in different therapeutic modalities. While our study primarily focused on extended psychotherapeutic interventions exceeding 8 weeks, it is essential to acknowledge the efficacy of psychodynamic psychotherapy with CYP, often requiring a longer duration. A recent systematic review by Midgley et al. (74) evaluated the evidence base for psychodynamic therapy. Their synthesis of findings highlights the effectiveness of psychodynamic therapy in addressing a broad range of MH challenges, particularly internalizing disorders like depression and anxiety, emerging personality disorders, and adversity-experienced children. Importantly, the review reveals that psychodynamic psychotherapy, a modality efficient in these contexts, frequently extends beyond the 8-week benchmark. This emphasizes the necessity of tailoring therapeutic durations based on specific goals and individual needs.

## 5 Strengths and weaknesses

This systematic review has several strengths. We used *a priori* library search strategy with the use of six databases, with well-defined inclusion and exclusion criteria for the studies. The screening process, as well as the evaluation of risk of bias and NHMRC assessment, involved independent reviewers, ensuring rigorous methodology.

In considering the limitations of this study, it is important to acknowledge that certain studies were classified under multiple categories. This suggests interrelatedness rather than complete independence, as delineated in the text (75). There was also a lack of standardized interventions and outcomes, which precluded meta-analysis. Additionally, we only included studies published in English and did not explore the grey literature. Further high-level, high-quality research using standardized outcome measures is required to support these findings and determine key parameters, such as an optimal frequency and duration for long term intervention programs.

While conducting this systematic review, there is the potential bias associated with encompassing the term CYP. Adolescence, representing a distinct phase marked by biological, social, and role transitions, challenges the homogeneity of the term ‘CYP’ (76). Acknowledging the inherent differences between children and adolescents is crucial, both in clinical practice and research. Sawyer et al. (76) propose an expanded definition of adolescence up to the age of 24, aligning more closely with the extended period of growth and social role transitions. Despite employing the term ‘CYP’ in line with common usage, it is essential to recognize the potential heterogeneity within this broad categorization. Future research should explore age-specific nuances in MH interventions, contributing to more targeted and effective interventions.

### 5.1 Recommendations

There is a need for high-quality, large-scale RCT studies to discern the effectiveness of interventions with long term follow-up data. For example, we presented outcomes associated with parenting programs, however as the studies were of low-quality evidence, a RCT would be required to substantiate the efficacy of the programs. Further, as most studies had well defined, protocol-driven, and well-supervised care (32), evidence for scaling-up and sustainability of MH interventions in lower sociodemographic areas needs to be strengthened (27). Additionally, we did not review any health economic outcomes, further research would be required to determine the cost-effectiveness of different long-term interventions across a wide spectrum of MH symptoms, severity levels, and age groups.

Future research into the benefits of long-term MH interventions, would be required to evaluate the effects of each intervention with a comparison group with consistent and standardized measurement tools, in addressing the specific outcomes of interest.

## 6 Conclusion

This systematic review focused on evaluating the impact of long-term interventions designed for CYP experiencing both acute and long-term MH crises. The findings offer insights to inform the development of comprehensive interventions for CYP with MH concerns and their families/caregivers. Our results demonstrate that collaborative care services, CBT, family group-focused interventions, MST, and parent training programs are effective in enhancing MH outcomes for CYP across various outpatient settings, including community services and in-home care. Importantly, these interventions have shown promising reductions in hospital readmission rates, suggesting their potential to mitigate the need for acute inpatient care.

However, the feasibility and acceptability of interventions with lower quality evidence among CYP and their families/caregivers demand further research. Rigorous studies incorporating pre-intervention comparison groups are essential to assess the effectiveness of these interventions in reducing symptomatology and improving MH function and quality of life across diverse MH symptoms, severity levels, and age groups. To enhance MH outcomes for CYP, interventions should prioritize patient safety, seamless care integration, and service quality, while also emphasizing consistent evaluation of novel interventions and therapies. Given the inherently long-term nature of these interventions, sustained effectiveness and success need to be evaluated through comprehensive, extended follow-up studies, ensuring an understanding of their impact over time.

## Data availability statement

The original contributions presented in the study are included in the article/**Supplementary Material**, further inquiries can be directed to the corresponding author.

## Author contributions

BG: Conceptualization, Data curation, Formal analysis, Investigation, Methodology, Visualization, Writing – original draft, Writing – review & editing. BA: Data curation, Formal analysis, Investigation, Methodology, Visualization, Writing – review & editing. TN: Data curation, Formal analysis, Investigation, Methodology, Visualization, Writing – review & editing. JJ: Data curation, Formal analysis, Investigation, Methodology, Visualization, Writing – review & editing. PH: Conceptualization, Data curation, Formal analysis, Investigation, Methodology, Visualization, Writing – review & editing. TW: Data curation, Formal analysis, Investigation, Methodology, Visualization, Writing – review & editing. FB: Data curation, Formal analysis, Investigation, Methodology, Visualization, Writing – review & editing. MB: Conceptualization, Funding acquisition, Investigation, Methodology, Project administration, Supervision, Writing – original draft, Writing – review & editing. VE: Conceptualization, Funding acquisition, Investigation, Methodology, Project administration, Supervision, Writing – original draft, Writing – review & editing.
