# Supplementary material for: Evidence-based long term interventions targeting acute mental health presentations for children and adolescents: systematic review
Source: Front Psychiatry. 2024 Mar 6;15:1324220. doi: 10.3389/fpsyt.2024.1324220 (PMC10950959; doi:10.3389/fpsyt.2024.1324220)
Supplement: Supplementary file 1 [file DataSheet_1.docx]

**Appendices 1-3**

**Appendix 1**

**Risk of bias**

The Cochrane tool for assessing risk of bias for RCTs (ROB-2) (69) and the Methodological Index for Non-randomised Studies (MINORS) score, with a global ideal score of 16 for non-comparative studies and 24 for comparative studies were used to assess risk of bias (70). Two reviewers (BOA, TN) independently assessed the risk of bias for each of the included studies. Any discrepancies were resolved by a third person (BG) acting as a moderator.

Following an assessment of each of the thirty studies using the MINORS tool, 16 studies were assessed as having a high risk of bias (33,36,39,40,44-49,52,53,56,57,60,61) and 7 as having a moderate risk (34,35,37,38,43,50,54). Among the seven RCTs which were assessed for their risk of bias using the ROB2, one study was deemed to have a low risk of bias (42), four studies had some concerns (moderate risk of bias) (32,55,58,59) and two studies were deemed to have a high risk of bias (41,51).

**Appendix 2**

***Quality assessment and data extraction***

The National Health and Medical Research Council (NHMRC) levels of evidence and grade guidelines tool was used by three reviewers (BG, PH, BOA) to independently assess the quality of evidence of included studies (69). The NHMRC levels of evidence use four levels of quality that constitute the Evidence Hierarchy (‘levels of evidence’ that are derived according to the type of research question within each study), with levels I (systematic reviews of randomised controlled trials [RCTs]), II (RCTs) and III-I (pseudo RCTs) graded as ‘higher’ level scores, elucidating greater confidence associated with the findings presented in each study. Further, level III-2 (comparative studies with controls such as non-randomised experimental trials, cohort studies, case-control studies, or interrupted time series with a control group) was graded as ‘a Moderate’ level of evidence. Levels III-3 (comparative studies without controls such as historical control study, two or more single-arm studies, interrupted time series without a parallel control group), and IV (case studies with post-test or pre and post-tests) were indicative of ‘lower levels’ of evidence. Discrepancies were resolved through discussion to reach a consensus.

**Appendix 3: Search Strategy**

| **Concept** | **Database** | | | | | | |  |
| --- | --- | --- | --- | --- | --- | --- | --- | --- |
|  | **Embase Classic + Embase**  **<2000 to 2022>** | | **Ovid Psychinfo**  **<2000-2022>** | | **PUBMED**  **<2000 to 2022>** | | **Web of Science**  **<2000-2022>** | **Cochrane**  **<2000-2022>** |
|  | **Free text terms** | **Mesh terms** | **Free text terms** | **Mesh terms** | **Free text terms** | **Mesh terms** | **Free text terms** | **Free text terms** |
| Young people aged 0-17 years | child*.mp.  adolesc*.mp.  youth*.mp.  p?ediatric*.mp.  teen*.mp.  pubert*.mp.  young.mp. | adolescent/ or child/  pediatrics/ or pediatric emergency medicine/  pediatrics/ or pediatric crisis  pediatrics/ or pediatric crisis intervention  young people.mp.  young person.mp.  exp Child Psychiatry/ exp Child Psychology  exp Adolescent Psychiatry/ or exp Adolescent Psychotherapy/ or exp Adolescent Psychology/  pediatrics/ or chronically ill children/ | child*.mp.  adolesc*.mp.  youth*.mp.  p?ediatric*.mp.  teen*.mp. | adolescent/ or child/  pediatrics/ or pediatric emergency medicine/  pediatrics/ or pediatric crisis  pediatrics/ or pediatric crisis intervention  young people.mp.  exp Child Psychiatry/ or exp Child Psychotherapy/ or exp Child Psychology/  exp Adolescent Psychiatry/ or exp Adolescent Psychotherapy/ or exp Adolescent Psychology/  pediatrics/ or chronically ill children/ | child*.mp.  adolesc*.mp.  youth*.mp.  p?ediatric*.mp.  teen*.mp.  young people.mp. | adolescent/ or child/  pediatrics/or pediatric emergency medicine/  pediatrics/ or pediatric crisis  pediatrics/ or pediatric crisis intervention | child*  adolesc*  youth*  paediatric*  teen*  young people  young person  “Child Psychology”  “Child Psychotherapy”  “Adolescent Psychiatry”  “Adolescent Psychotherapy”  “Adolescent Psychology”  “chronically ill child*” | child*  adolesc*  youth*  paediatric*  teen*  pubert*  young people  young person  “Child Psychology”  “Child Psychotherapy”  “Adolescent Psychiatry”  “Adolescent Psychotherapy”  “Adolescent Psychology”  “chronically ill child*” |
| Mental Illness | (mental adj3 problem*).mp.  mental health*    mental*  (mental adj3 cris?s).mp.  psychiatr*,mp.  mental illness*.mp.  somat*.mp.  malinger*.mp.  autis*.mp.  eating disorder*.mp.  rumination disorder*.mp.  abnormal behavio?r*.mp.  unusual behavio?r*.mp.  “substance use disorder”/  unexplained behavio?r*.mp.  unexplained symptom*.mp.  self?harm.mp.  suicid*.mp.  substance ?use.mp.  alcohol*.mp.  cannab*.mp.  overdose*.mp.  mental disorder*.mp. | anorexia.mp.  bulimia.mp.  purging.mp.  restrictive food intake.mp.  pica.mp.  drug abuse.mp.  alcohol abuse.mp.  psychosomatic.mp.  Substance-Related Disorders/  mental disorders/ or neurotic disorders/ or obsessive-compulsive disorder/ or panic disorder/ or "bipolar and related disorders"/ or "disruptive, impulse control, and conduct disorders"/ or dissociative disorders/ or "feeding and eating disorders"/ or amnesia/ or cognition disorders/ or "attention deficit and disruptive behavior disorders"/ or child behavior disorders/ or child development disorders, pervasive/ or schizophrenia, childhood/ or personality disorders/ or "schizophrenia spectrum and other psychotic disorders"/ or somatoform disorders/ or substance-related disorders/ or "trauma and stressor related disorders"/  exp Mental Disorders/  mental disease/  mental disease.mp. or exp mental disease/  attempted suicide/  exp Eating Disorders/  exp Somatoform Pain Disorder/ or exp Somatoform Disorders/ | (mental adj3 problem*).mp.  (mental adj3 cris?s).mp.  psychiatr*.mp.  mental illness*.mp.  somat*.mp.  malinger*.mp.  autis*.mp.  eating disorder*.mp.  rumination disorder*.mp.  abnormal behavio?r*.mp.  unusual behavio?r*.mp.  unexplained behavio?r*.mp.  unexplained symptom*.mp.  self?harm.mp.  suicid*.mp.  substance ?use.mp.  alcohol*.mp.  cannab*.mp.  overdose*.mp.  mental disorder*.mp.  "substance use disorder"/ | mental disease.mp. or exp mental disease/  psychosomatic.mp.  anorexia.mp.  bulimia.mp.  purging.mp.  restrictive food intake.mp.  pica.mp.  drug abuse.mp.  Substance-Related Disorders/  mental disorders/ or neurotic disorders/ or obsessive-compulsive disorder/ or panic disorder/ or "bipolar and related disorders"/ or "disruptive, impulse control, and conduct disorders"/ or dissociative disorders/ or "feeding and eating disorders"/ or amnesia/ or cognition disorders/ or "attention deficit and disruptive behavior disorders"/ or child behavior disorders/ or child development disorders, pervasive/ or schizophrenia, childhood/ or personality disorders/ or "schizophrenia spectrum and other psychotic disorders"/ or somatoform disorders/ or substance-related disorders/ or "trauma and stressor related disorders"/  exp Mental Disorders/  attempted suicide/  exp Eating Disorders/  exp Somatoform Pain Disorder/ or exp Somatoform Disorders/  mental disease/ | (mental adj3 problem*).mp.  (mental adj3 cris?s).mp.  psychiatr*.mp.  mental illness*.mp.  somat*.mp.  psychosomatic.mp.  malinger*.mp.  autis*.mp.  binge?eating.mp.  rumination disorder*.mp.  abnormal behavio?r*.mp.  unusual behavio?r*.mp.  unexplained behavio?r*.mp.  unexplained symptom*.mp.  self?harm.mp.  suicid*.mp.  substance ?use.mp.  alcohol*.mp.  cannab*.mp.  overdose*.mp. | anorexia.mp.  bulimia.mp.  purging.mp.  restrictive food intake.mp.  pica.mp.  drug abuse.mp  Substance-Related Disorders/  mental disorders/ or “bipolar and related disorders” /or “disruptive, impulse control, and conduct disorders”/ or dissociative disorders/ or “feeding and eating disorders”/ or amnesia/ or cognition disorders/ or “attention deficit and disruptive behaviour disorders”/ or child behaviour disorders/ or child development disorders, pervasive/ or schizophrenia, childhood/ or neurotic disorders/ or somatoform disorders/ or substance-related disorders/ or “trauma and stressor related disorders” /or “behavioural disciplines and activities”/ | mental near/2 problem*  mental near/2 cris*  psychiatr*  mental illness*  somat*  “psychosomatic”  alcohol*  alcohol abuse.mp.  malinger*  autis*  anorexia  purging  “eating disorder”  “restrictive food intake”  “rumination disorder”  pica  “abnormal behaviour*”  “unusual behaviour*”  “abnormal behavior*”  “unusual behavior*”  “unexplained behaviour*”  “unexplained behavior*”  “unexplained symptom*”  “self harm”  self-harm  suicid*  “substance use*”  “substance abuse”  alcohol*  cannab*  overdose*  “drug abuse”  “substance-related disorders”  “neurotic disorder*”  “mental disorder*”  “obsessive-compulsive disorder”  “panic disorder”  “bipolar and related disorders”  “disruptive, impulse control and conduct disorders”  “feeding and eating disorders”  amnesia  “cognition disorders”  “attention deficit and disruptive behavior disorders”  “child behavior disorders”  “attention deficit and disruptive behaviour disorders”  “dissociative disorder*”  “child behaviour disorders”  “child development disorders, pervasive”  schizophrenia, childhood  personality disorders  “schizophrenia spectrum and other psychotic disorders”  “somatoform disorder*”  “substance-related disorders”  “trauma and stressor related disorders”  bulimi*  “binge eating”  “behavioural disciplines and activities”  “attempted suicide”  “somatoform pain disorder” | mental near/2 problem*  mental near/2 cris*  psychiatr*  mental illness*  somat*  “psychosomatic”  malinger*  alcohol*  alcohol abuse.mp.  autis*  anorexia  purging  “eating disorder”  “restrictive food intake”  “rumination disorder”  pica  “abnormal behaviour*”  “unusual behaviour*”  “abnormal behavior*”  “unusual behavior*”  “unexplained behaviour*”  “unexplained behavior*”  “unexplained symptom*”  “self harm”  self-harm  suicid*  “substance use*”  “substance abuse”  alcohol*  cannab*  overdose*  “drug abuse”  “substance-related disorders”  “neurotic disorder*”  “mental disorder*”  “obsessive-compulsive disorder”  “panic disorder”  “bipolar and related disorders”  “disruptive, impulse control and conduct disorders”  “feeding and eating disorders”  amnesia  “cognition disorders”  “attention deficit and disruptive behavior disorders”  “child behavior disorders”  “attention deficit and disruptive behaviour disorders”  “dissociative disorder*”  “child behaviour disorders”  “child development disorders, pervasive”  schizophrenia, childhood  personality disorders  “schizophrenia spectrum and other psychotic disorders”  “somatoform disorder*”  “substance-related disorders”  “trauma and stressor related disorders”  bulimi*  “binge eating”  “behavioural disciplines and activities”  “attempted suicide”  “somatoform pain disorder” |
| Intervention | famil*.mp.  cognitive* behav*.mp.  dialect* behav*.mp.  well*.mp.  counsel*.mp.  family therapy.mp.  individ*consel*.mp.  psycho* therapy*.mp.  parent-child dyad  healthcare* deliv*.mp.  invidi* counsel*.mp  parent*therap*.mp.  school based therp*.mp.  psycho care.mp.  group session*.mp  group thera*.mp.  counsel*.mp.  care co-ordin*.mp.  home* care.mp.  patient cent* care.mp.  community based care.mp.  holistic care.mp.  safety care.mp.  risk assessmen*.mp.  In hospital*.mp.  drug counsel*.mp.  pharmacotherapy*.mp.  medication manage*.mp.  assessment serv*.mp.  model of care.mp.  delivery of care.mp.  intensive interven*.mp.  medical home care.mp.  integr* care.mp.  integrated care*  emergency admission*.mp.  school*.mp.  wrap-around care*.mp.  emergency readmission*.mp.  rehospitali*.mp.  (hospital adj3 emergenc*).mp.  (emergenc* adj2 medic*).mp.  acute hospital*.mp.  (acute adj2 medic*).mp.  (emergenc* adj2 treatment*).mp.  re?feeding.mp.  (emergenc* adj2 in?patient).mp.  (non?elective adj2 care).mp.  (non?elective adj2 treatment).mp.  (unscheduled adj2 care).mp.  (unscheduled adj2 treatment).mp.  (unscheduled adj2 medical).mp.  (non?elective adj2 medical).mp.  (unplanned adj2 care).mp.  (unplanned adj2 treatment).mp.  (unplanned adj2 medical).mp.  (prospective adj2 care).mp.  (prospective adj2 treatment).mp.  (prospective adj2 medical).mp.  (urgent adj2 care).mp.  (urgent adj2 treatment).mp.  (urgent adj2 medical).mp.  emergency hospitali?ation*.mp.  acute hospitali?ation.mp.  acute treatment*.mp.  acute admission*.mp. | famil*.mp.  cognitive* behav*.mp.  dialect* behav*.mp.  well*.mp.  counsel*.mp.  family therapy.mp.  individ*consel*.mp.  psycho* therapy*.mp.  parent-child dyad  healthcare* deliv*.mp.  invidi* counsel*.mp  parent*therap*.mp.  school based therp*.mp.  psycho care.mp.  intensive interv*.mp.  group session*.mp.  group thera*.mp.  counsel*.mp.  care co-ordin*.mp.  home* care.mp.  patient cent* care.mp.  community based care.mp.  holistic care.mp.  safety care.mp.  risk assessmen*.mp.  In hospital*.mp.  drug counsel*.mp.  pharmacotherapy*.mp.  medication manage*.mp.  assessment serv*.mp.  model of care.mp.  delivery of care.mp.  medical home care.mp.  integr* care.mp.  integrated care*  emergency admission*.mp.  school*.mp.  wrap-around care*.mp.  emergency medicine/ or pediatric emergency medicine/  emergency medicine/  emergency treatment/  acute care.mp.  acute ward.mp.  emergency services/ or crisis intervention services/ or emergency medicine/  hospital emergency service/ or emergency health service/ | famil*.mp.  cognitive* behav*.mp.  dialect* behav*.mp.  well*.mp.  counsel*.mp.  family therapy.mp.  individ*consel*.mp.  psycho* therapy*.mp.  parent-child dyad  healthcare* deliv*.mp.  invidi* counsel*.mp  parent*therap*.mp.  school based therp*.mp.  psycho care.mp.  group session*.mp.  group thera*.mp.  counsel*.mp.  care co-ordin*.mp.  home* care.mp.  patient cent* care.mp.  community based care.mp.  holistic care.mp.  safety care.mp.  risk assessmen*.mp.  In hospital*.mp.  drug counsel*.mp.  pharmacotherapy*.mp.  medication manage*.mp.  assessment serv*.mp.  model of care.mp.  delivery of care.mp.  medical home care.mp.  integr* care.mp.  integrated care*  emergency admission*.mp.  school*.mp.  wrap-around care*.mp.  emergency readmission*.mp.  wrap-around care*.mp.  rehospitali*.mp.  (hospital adj3 emergenc*).mp.  (emergenc* adj2 medic*).mp.  acute hospital*.mp.  (acute adj2 medic*).mp.  (emergenc* adj2 treatment*).mp.  re?feeding.mp.  (emergenc* adj2 in?patient).mp.  (non?elective adj2 care).mp.  (non?elective adj2 treatment).mp.  (unscheduled adj2 care).mp.  (unscheduled adj2 treatment).mp.  (unscheduled adj2 medical).mp.  (non?elective adj2 medical).mp.  (unplanned adj2 care).mp.  (unplanned adj2 treatment).mp.  (unplanned adj2 medical).mp.  (prospective adj2 care).mp.  (prospective adj2 treatment).mp.  (prospective adj2 medical).mp.  (urgent adj2 care).mp.  (urgent adj2 treatment).mp.  (urgent adj2 medical).mp.  emergency hospitali?ation*.mp.  acute hospitali?ation.mp.  acute treatment*.mp.  acute admission*.mp. | famil*.mp.  cognitive* behav*.mp.  dialect* behav*.mp.  well*.mp.  counsel*.mp.  family therapy.mp.  individ*consel*.mp.  psycho* therapy*.mp.  parent-child dyad  healthcare* deliv*.mp.  invidi* counsel*.mp  parent*therap*.mp.  school based therp*.mp.  psycho care.mp.  group session*.mp.  intensive interv*.mp.  group thera*.mp.  counsel*.mp.  care co-ordin*.mp.  home* care.mp.  patient cent* care.mp.  community based care.mp.  holistic care.mp.  safety care.mp.  risk assessmen*.mp.  In hospital*.mp.  drug counsel*.mp.  pharmacotherapy*.mp.  medication manage*.mp.  assessment serv*.mp.  model of care.mp.  delivery of care.mp.  medical home care.mp.  integr* care.mp.  integrated care*  emergency admission*.mp.  school*.mp.  wrap-around care*.mp.  emergency medicine/ or pediatric emergency medicine/  acute care.mp.  acute ward.mp.  emergency services/ or crisis intervention services/or emergency medicine/  hospital emergency service/ or emergency health service/  emergency medicine/  emergency treatment/ | famil*.mp.  cognitive* behav*.mp.  dialect* behav*.mp.  well*.mp.  counsel*.mp.  family therapy.mp.  individ*consel*.mp.  psycho* therapy*.mp.  parent-child dyad  healthcare* deliv*.mp.  invidi* counsel*.mp  parent*therap*.mp.  school based therp*.mp.  psycho care.mp.  group session*.mp.  group thera*.mp.  counsel*.mp.  care co-ordin*.mp.  home* care.mp.  patient cent* care.mp.  intensive interv*.mp.  community based care.mp.  holistic care.mp.  safety care.mp.  risk assessmen*.mp.  In hospital*.mp.  drug counsel*.mp.  pharmacotherapy*.mp.  medication manage*.mp.  assessment serv*.mp.  model of care.mp.  delivery of care.mp.  medical home care.mp.  integr* care.mp.  integrated care*  emergency admission*.mp.  school*.mp.  wrap-around care*.mp.  emergency admission*.mp.  wrap-around care*.mp.  emergency readmission*.mp.  rehospitali*.mp.  (hospital* adj4 emergenc*).mp.  (emergenc* adj2 medic*).mp.  acute hospital*.mp.  (acute adj2 medic*).mp.  (emergenc* adj2 treatment*).mp.  medical stabili?ation.mp  re?feeding.mp.  (acute adj2 in?patient*).mp.  (emergenc* adj2 in?patient*).mp.  (hospital adj2 readmi*).mp.  (non?elective adj2 care).mp.  (non?elective adj2 admission*).mp.  (non?elective adj2 treatment).mp.  (non?elective adj2 readmission*).mp.  (unscheduled adj2 care).mp.  (unscheduled adj2 admission*).mp.  (unscheduled adj2 readmission*).mp.  (unscheduled adj2 treatment).mp.  (unscheduled adj2 hospital*).mp.  (unplanned adj2 hospital*).mp.  (unplanned adj2 admi*).mp.  (unplanned adj2 care).mp.  (unplanned adj2 treatment).mp.  (unplanned adj2 readmi*).mp.  (non?elective adj2 hospital*).mp  (prospective adj2 care).mp.  (prospective adj2 admission*).mp.  (prospective adj2 treatment).mp.  (prospective adj2 readmission*).mp.  (urgent adj2 care).mp.  (urgent adj2 admission*).mp.  (urgent adj2 readmission*).mp.  (urgent adj2 treatment).mp. | famil*.mp.  cognitive* behav*.mp.  dialect* behav*.mp.  well*.mp.  counsel*.mp.  family therapy.mp.  individ*consel*.mp.  psycho* therapy*.mp.  parent-child dyad  healthcare* deliv*.mp.  invidi* counsel*.mp  parent*therap*.mp.  school based therp*.mp.  psycho care.mp.  intensive interv*.mp.  group session*.mp.  group thera*.mp.  counsel*.mp.  care co-ordin*.mp.  home* care.mp.  patient cent* care.mp.  community based care.mp.  holistic care.mp.  safety care.mp.  risk assessmen*.mp.  In hospital*.mp.  drug counsel*.mp.  pharmacotherapy*.mp.  medication manage*.mp.  assessment serv*.mp.  model of care.mp.  delivery of care.mp.  medical home care.mp.  integr* care.mp.  integrated care*  emergency admission*.mp.  school*.mp.  wrap-around care*.mp.  emergency medicine/ or pediatric emergency medicine/  well*.mp.  counsel*.mp.  family therapy.mp.  individ*consel*.mp.  psycho* therapy*.mp.  healthcare* deliv*.mp.  parent-child dyad  acute care.mp.  Emergency Service, Hospital  Child, Hospitalized/ or Adolescent, Hospitalized/  hospitalisation/ or “length of stay” / or patient admission/ or patient readmission | famil*.mp.  cognitive* behav*.mp.  dialect* behav*.mp.  well*.mp.  counsel*.mp.  family therapy.mp.  individ*consel*.mp.  psycho* therapy*.mp.  parent-child dyad  healthcare* deliv*.mp.  invidi* counsel*.mp  parent*therap*.mp.  school based therp*.mp.  psycho care.mp.  group session*.mp.  group thera*.mp.  counsel*.mp.  care co-ordin*.mp.  home* care.mp.  intensive interv*.mp.  patient cent* care.mp.  community based care.mp.  holistic care.mp.  safety care.mp.  risk assessmen*.mp.  In hospital*.mp.  drug counsel*.mp.  pharmacotherapy*.mp.  medication manage*.mp.  assessment serv*.mp.  model of care.mp.  delivery of care.mp.  medical home care.mp.  integr* care.mp.  integrated care*  emergency admission*.mp.  school*.mp.  wrap-around care*.mp.  hospital near/2 emergenc*  healthcare* deliv*.mp.  well*.mp.  counsel*.mp.  family therapy.mp.  individ*consel*.mp.  psycho* therapy*.mp.  parent-child dyad  emergenc* near/1 medic*  “acute hospital*”  “emergency service, hospital”  “emergency medicine”  “pediatric emergency medicine”  emergenc* near/1 treatment*  “medical stabil?ation”  refeeding  emergenc* near/1 inpatient*  nonelective near/1 care  nonelective near/1 treatment  unscheduled near/1 medical  nonelective near/1 medical  unplanned near/1 care  unplanned near/1 treatment  acute near/1 medic*  unscheduled near/1 care  unscheduled near/1 treatment  unplanned near/1 medical  prospective near/1 care  prospective near/1 treatment  prospective near/1 medical  urgent near/1 care  urgent near/1 treatment  urgent near/1 medical  hospitali*  length of stay near/2 acute  acute near/1 inpatient*  hospital near/1 readmi*  nonelective near/1 admission*  nonelective near/1 readmission*  unscheduled near/1 admission*  unscheduled near/1 readmission*  unscheduled near/1 hospital*  unplanned near/1 hospital*  unplanned near/1 admi*  unplanned near/1 readmi*  nonelective near/1 hospital*  Child, Hospitalised  Adolescent, Hospitalised  prospective near/1 admission*  prospective near/1 readmission*  urgent near/1 admission*  urgent near/1 readmission*  “emergency hospitalisation*”  “emergency hospitalization*”  “acute hospitalisation*”  “acute hospitalization*”  “acute treatment*”  “acute admission*”  “acute ward”  “emergency services”  “crisis intervention services” | famil*.mp.  cognitive* behav*.mp.  dialect* behav*.mp.  well*.mp.  counsel*.mp.  family therapy.mp.  individ*consel*.mp.  psycho* therapy*.mp.  parent-child dyad  healthcare* deliv*.mp.  invidi* counsel*.mp  parent*therap*.mp.  school based therp*.mp.  psycho care.mp.  group session*.mp.  group thera*.mp.  counsel*.mp.  care co-ordin*.mp.  home* care.mp.  patient cent* care.mp.  community based care.mp.  holistic care.mp.  safety care.mp.  intensive interv*.mp.  risk assessmen*.mp.  In hospital*.mp.  drug counsel*.mp.  pharmacotherapy*.mp.  medication manage*.mp.  assessment serv*.mp.  model of care.mp.  delivery of care.mp.  medical home care.mp.  integr* care.mp.  integrated care*  emergency admission*.mp.  school*.mp.  wrap-around care*.mp.  “acute care”  “emergency admission*”  school*.mp.  “emergency readmission*”  rehospitali*  hospital near/2 emergenc*  wrap-around care*.mp.  healthcare* deliv*.mp.  emergenc* near/1 medic*  well*.mp.  counsel*.mp.  family therapy.mp.  individ*consel*.mp.  psycho* therapy*.mp.  parent-child dyad  “acute hospital*”  “emergency service, hospital”  “emergency medicine”  “pediatric emergency medicine”  emergenc* near/1 treatment*  “medical stabil?ation”  refeeding  emergenc* near/1 inpatient*  nonelective near/1 care  nonelective near/1 treatment  unscheduled near/1 medical  nonelective near/1 medical  unplanned near/1 care  unplanned near/1 treatment  acute near/1 medic*  unscheduled near/1 care  unscheduled near/1 treatment  unplanned near/1 medical  prospective near/1 care  prospective near/1 treatment  prospective near/1 medical  urgent near/1 care  urgent near/1 treatment  urgent near/1 medical  hospitali*  length of stay near/2 acute  acute near/1 inpatient*  hospital near/1 readmi*  nonelective near/1 admission*  nonelective near/1 readmission*  unscheduled near/1 admission*  unscheduled near/1 readmission*  unscheduled near/1 hospital*  unplanned near/1 hospital*  unplanned near/1 admi*  unplanned near/1 readmi*  nonelective near/1 hospital*  Child, Hospitalised  Adolescent, Hospitalised  prospective near/1 admission*  prospective near/1 readmission*  urgent near/1 admission*  urgent near/1 readmission*  “emergency hospitalisation*”  “emergency hospitalization*”  “acute hospitalisation*”  “acute hospitalization*”  “acute treatment*”  “acute admission*”  “acute ward”  “emergency services”  “crisis intervention services” |

**References**

1. Sterne JA, Savović J, Page MJ, Elbers RG, Blencowe NS, Boutron I, et al. RoB 2: a revised tool for assessing risk of bias in randomised trials. bmj. 2019;366.

2. Slim K, Nini E, Forestier D, Kwiatkowski F, Panis Y, Chipponi J. Methodological index for non‐randomized studies (MINORS): development and validation of a new instrument. ANZ journal of surgery. 2003;73(9):712-6.

3. Baruch G, Vrouva I, Wells C. Outcome Findings from a Parent Training Programme for Young People with Conduct Problems. Child and Adolescent Mental Health. 2011;16:47-54.

4. Clossey L, Simms S, Hu C, Hartzell J, Duah P, Daniels L. A pilot evaluation of the rapid response program: A home based family therapy. Community Mental Health Journal. 2018;54(3):302-11.

5. Duffy F, Skeldon J. A CAMHS Intensive Treatment Service: Clinical outcomes in the first year. Clinical Child Psychology and Psychiatry. 2012;19(1):90-9.

6. Flynn D, Kells M, Joyce M, Corcoran P, Gillespie C, Suarez C, et al. Innovations in Practice: Dialectical behaviour therapy for adolescents: multisite implementation and evaluation of a 16-week programme in a public community mental health setting. Child and Adolescent Mental Health. 2019;24:76-83.

7. Fox RA, Holtz CA. Treatment outcomes for toddlers with behaviour problems from families in poverty. Child and Adolescent Mental Health. 2009;14:183-9.

8. Griffiths H, Noble A, Duffy F, Schwannauer M. Innovations in Practice: Evaluating clinical outcome and service utilization in an AMBIT-trained Tier 4 child and adolescent mental health service. Child and Adolescent Mental Health. 2017;22:170-4.

9. James AC, Winmill L, Anderson C, Alfoadari K. A Preliminary Study of an Extension of a Community Dialectic Behaviour Therapy (DBT) Programme to Adolescents in the Looked After Care System. Child and Adolescent Mental Health. 2011;16:9-13.

10. Lilly RG, Meadows TJ, Sevecke-Hanrahan JR, Massura CE, Golden ME, O'Dell SM. Hub-extension model and access to pediatric behavioral integrated primary care. Clinical Practice in Pediatric Psychology. 2020;8(3):220-7.

11. Lu ZQ, de Geus H, Roest S, Payne L, Krishnamoorthy G, Littlewood R, et al. Characteristics and treatment outcomes of children and adolescents accessing treatment in Child and Youth Mental Health Services. EARLY INTERVENTION IN PSYCHIATRY. 2022.

12. Mantzouranis G, Baier V, Holzer L, Urben S, Villard E. Clinical significance of assertive community treatment among adolescents. J Social Psychiatry. 2019;54(4):445-53.

13. McDonell MG, Tarantino J, Dubose AP, Matestic P, Steinmetz K, Galbreath H, et al. A Pilot Evaluation of Dialectical Behavioural Therapy in Adolescent Long-Term Inpatient Care. Child and Adolescent Mental Health. 2010;15:193-6.

14. Newman M, Fagan C, Webb R. Innovations in Practice: The efficacy of nonviolent resistance groups in treating aggressive and controlling children and young people: A preliminary analysis of pilot NVR groups in Kent. Child and Adolescent Mental Health. 2014;19:138-41.

15. Rickwood DJ, Mazzer KR, Telford NR, Parker AG, Tanti CJ, McGorry PD. Changes in psychological distress and psychosocial functioning in young people visiting headspace centres for mental health problems. Medical Journal of Australia. 2015;202(10):537-42.

16. Sibley MH, Olson S, Morley C, Campez M, Pelham WE. A school consultation intervention for adolescents with ADHD: barriers and implementation strategies. Child and Adolescent Mental Health. 2016;21:183-91.

17. Simpson W, Cowie L, Wilkinson L, Lock N, Monteith G. The Effectiveness of a Community Intensive Therapy Team on Young People's Mental Health Outcomes. Child and Adolescent Mental Health. 2010;15:217-23.

18. Webster-Stratton C, Rinaldi J, Reid JM. Long-Term Outcomes of Incredible Years Parenting Program: Predictors of Adolescent Adjustment. Child and Adolescent Mental Health. 2011;16:38-46.

19. Cordell KD, Snowden LR. Reducing Mental Health Emergency Services for Children Served Through California's Full Service Partnerships. Medical Care. 2017;55:299-305.

20. Enns JE, Randall JR, Smith M, Chateau D, Taylor C, Brownell M, et al. A Multimodal Intervention for Children with ADHD Reduces Inequity in Health and Education Outcomes. Canadian Journal of Psychiatry. 2017;62:403-12.

21. Evans GD, Radunovich HL, Cornette MM, Wiens BA, Roy A. Implementation and utilization characteristics of a rural, school-linked mental health program. Journal of Child and Family Studies. 2008;17(1):84-97.

22. Fjermestad KW, Wergeland GJ, Rogde A, Bjaastad JF, Heiervang E, Haugland BSM. School-based targeted prevention compared to specialist mental health treatment for youth anxiety. Child and Adolescent Mental Health. 2020;25:102-9.

23. Haft SL, Chen T, LeBlanc C, Tencza F, Hoeft F. Impact of mentoring on socio-emotional and mental health outcomes of youth with learning disabilities and attention-deficit hyperactivity disorder. Child and Adolescent Mental Health. 2019;24:318-28.

24. Jaycox LH, Morral AR, Juvonen J. Mental health and medical problems and service use among adolescent substance users. Journal of the American Academy of Child and Adolescent Psychiatry. 2003;42:701-9.

25. Shippee ND, Mattson A, Brennan R, Huxsahl J, Billings ML, Williams MD. Effectiveness in regular practice of collaborative care for depression among adolescents: A retrospective cohort study. Psychiatric Services. 2018;69(5):536-41.

26. Richardson LP, Ludman E, McCauley E, Lindenbaum J, Larison C, Zhou C, et al. Collaborative care for adolescents with depression in primary care: a randomized clinical trial. Jama. 2014;312(8):809-16.

27. Asarnow JR, Jaycox LH, Tang L, Duan N, LaBorde AP, Zeledon LR, et al. Long-term benefits of short-term quality improvement interventions for depressed youths in primary care. American Journal of Psychiatry. 2009;166:1002-10.

28. Boege I, Corpus N, Weichard M, Schepker R, Young P, Fegert JM. Long-term outcome of intensive home treatment for children and adolescents with mental health problems - 4 years after a randomized controlled clinical trial. Child and Adolescent Mental Health. 2021;26:310-9.

29. Ogden T, Hagen KA. Multisystemic treatment of serious behaviour problems in youth: Sustainability of effectiveness two years after intake. Child and Adolescent Mental Health. 2006;11:142-9.

30. Van Den Hoofdakker BJ, Van Der Veen-Mulders L, Sytema S, Emmelkamp PMG, Minderaa RB, Nauta MH. Effectiveness of behavioral parent training for children with ADHD in routine clinical practice: A randomized controlled study. Journal of the American Academy of Child and Adolescent Psychiatry. 2007;46:1263-71.

31. Lipman EL, Boyle MH, Cunningham C, Kenny M, Sniderman C, Duku E, et al. Testing effectiveness of a community-based aggression management program for children 7 to 11 years old and their families. Journal of the American Academy of Child and Adolescent Psychiatry. 2006;45:1085-93.

32. Petrenko CLM, Pandolfino ME, Robinson LK. Findings from the Families on Track Intervention Pilot Trial for Children with Fetal Alcohol Spectrum Disorders and Their Families. Alcoholism: Clinical and Experimental Research. 2017;41:1340-51.

33. National Health and Medical Research Council (NHMRC) Working to build a Health Australia: NHMRC levels of evidence and grades for recommendations for developers of guidelines. December 2009 [

34. Popay J, Roberts H, Sowden A, Petticrew M, Arai L, Rodgers M, et al. Guidance on the conduct of narrative synthesis in systematic reviews. A product from the ESRC methods programme Version. 2006;1(1):b92.
